# Infective endocarditis of an aorto-right atrial fistula caused by asymptomatic rupture of a sinus of Valsalva aneurysm: a case report

**DOI:** 10.1186/s40792-016-0171-4

**Published:** 2016-05-15

**Authors:** Akihiko Ikeda, Tomomi Nakajima, Taisuke Konishi, Kanji Matsuzaki, Akinori Sugano, Yuko Fumikura, Hidetaka Nishina, Tomoaki Jikuya

**Affiliations:** Department of Cardiovascular Surgery, Tsukuba Medical Center Hospital, 1-3-1 Amakubo, Tsukuba, Ibaraki 305-8558 Japan; Department of Cardiology, Tsukuba Medical Center Hospital, Tsukuba, Ibaraki Japan

**Keywords:** Sinus of Valsalva aneurysm, Aorto-right atrial fistula, Infective endocarditis

## Abstract

Asymptomatic rupture of a sinus of Valsalva aneurysm is rare. A fistula following rupture of a sinus of Valsalva aneurysm may cause infective endocarditis. Here, we report a case of infective endocarditis of an aorto-right atrial fistula caused by asymptomatic rupture of a sinus of Valsalva aneurysm. A 45-year-old male, who was first diagnosed with a heart murmur at the age of 37 years, presented with fever. Blood culture was positive for *Streptococcus gordonii*. Ultrasound echocardiography revealed an aorto-right atrial fistula caused by rupture of a sinus of Valsalva aneurysm. After the infective endocarditis was healed by antibiotics, we successfully performed surgical repair of the aorto-right atrial fistula. Although asymptomatic rupture of a sinus of Valsalva aneurysm is uncommon, it should be recognized as a possible cause of infective endocarditis.

## Background

Sinus of Valsalva aneurysms can rupture into the right ventricle and atrium, causing an L-R shunt via a fistula. Rupture of a sinus of Valsalva aneurysm is usually accompanied by acute-onset symptoms [1]; however, the rupture is rarely asymptomatic [2, 3]. A fistula caused by rupture of a sinus of Valsalva aneurysm can cause infective endocarditis [4]. We experienced a case of infective endocarditis developing from asymptomatic rupture of a sinus of Valsalva aneurysm. The patient had remained asymptomatic for 8 years since the rupture of the sinus of Valsalva aneurysm, until the development of infective endocarditis.

## Case presentation

**Fig. 1:**
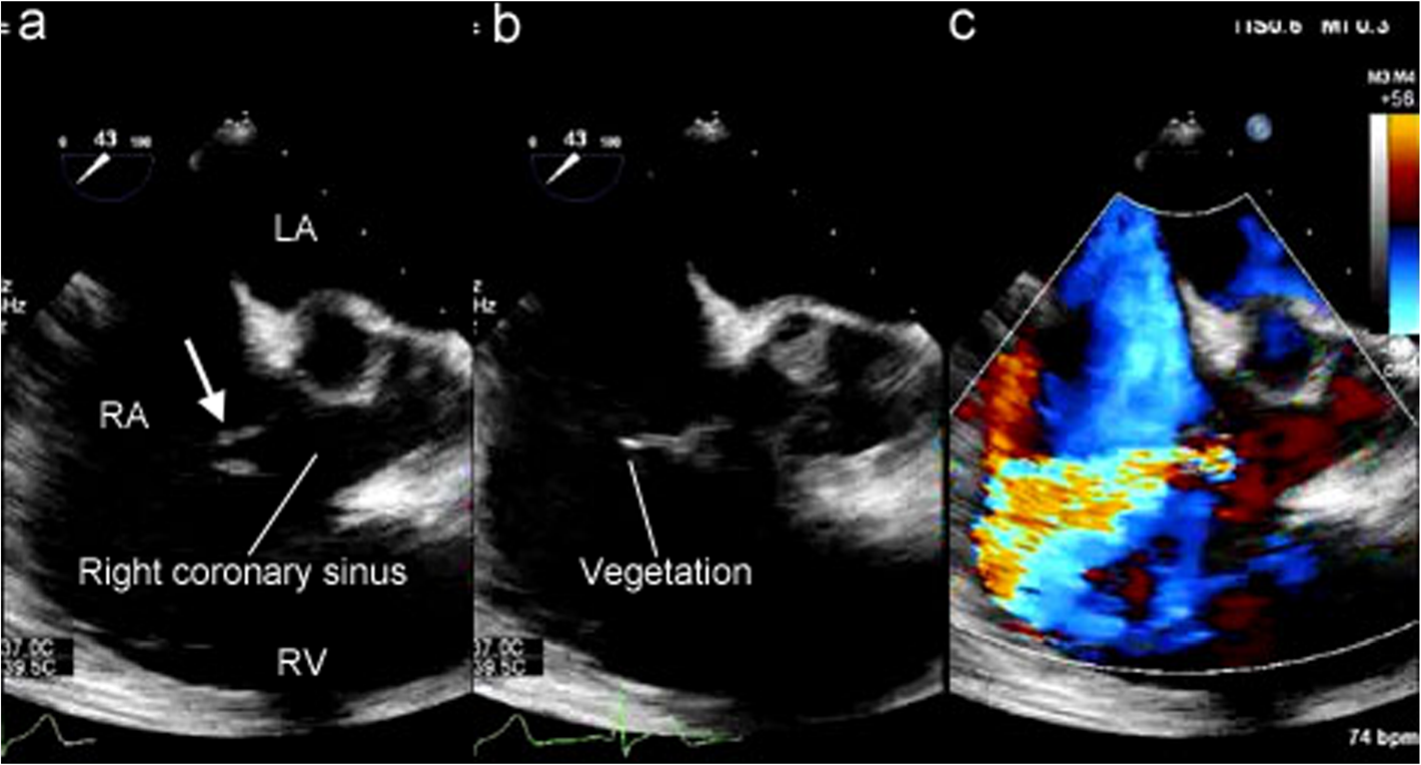
**a** Preoperative transesophageal echocardiography showing a windsock-like fistula tract originating in the right coronary sinus protruding into the right atrium (*arrow*). **b** String-shaped vegetation was attached to the tip of the windsock-like fistula. **c** An L-R shunt via the fistula tract was seen. *LA* left atrium, *RA* right atrium, *RV* right ventricle

**Fig. 2:**
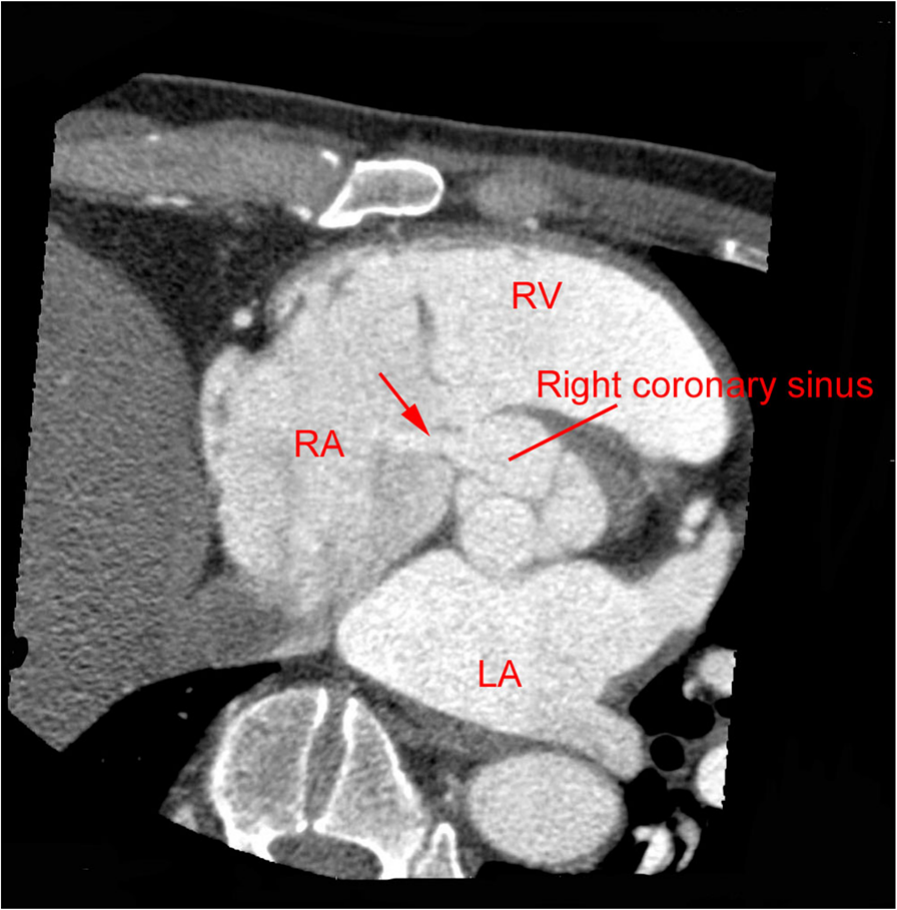
Preoperative computed tomography showing a defect between the right coronary sinus and the right atrium (*arrow*). *LA* left atrium, *RA* right atrium, *RV* right ventricle

A 45-year-old male was admitted to our hospital with a 3-month history of fever, general fatigue, and weight loss. Before visiting our hospital, he went to another hospital where he was administered antibiotics because his body temperature was over 38 °C. His significant past medical history included a heart murmur. He had first been diagnosed with a heart murmur at the age of 37 years but did not undergo further examinations. Although he had periodically received medical examinations while in school and at workplace, a heart murmur had not been diagnosed previously. There were no other remarkable past medical histories, including infection, trauma, or connective tissue disorders. On physical examination, his body temperature was 36.9 °C and no clinical signs of heart failure were observed. A continuous murmur was heard best at the right lower parasternal border. Chest X-ray did not reveal cardiomegaly or pulmonary edema. Laboratory examination revealed an elevated inflammatory response with a C-reactive protein (CRP) level of 3.9 mg/dl and anemia with a hemoglobin level of 10.7 g/dl. Two sets of blood cultures were positive for *Streptococcus gordonii*. Transesophageal echocardiography revealed a continuous L-R shunt from the right coronary sinus to the right atrium. The fistula tract was observed as a windsock deformity [5] originating in the right coronary sinus and projecting into the right atrium. String-shaped vegetation was attached to the tip of the windsock deformity (Fig. 1a–c). All heart valves were intact. Cardiac computed tomography confirmed a defect of the right coronary sinus, which resulted in a communication with the right atrium (Fig. 2). No abnormalities were found in the coronary arteries. The sinus of Valsalva was not dilated. Cardiac catheterization revealed the following values: right ventricle pressure, 35/6 mmHg; left ventricle pressure, 130/10 mmHg; and Qp/Qs, 1.8.

The patient was diagnosed with endocarditis of the aorto-right atrial fistula following rupture of a sinus of Valsalva aneurysm. We initiated antibiotic therapy because the hemodynamics and respiratory condition were stable. Ceftriaxone 2 g/day for 4 weeks and gentamicin 140 mg/day for 2 weeks were administered, and the infective endocarditis was healed; more specifically, CRP was normalized and the vegetation disappeared on echocardiography. Surgery was scheduled for the aorto-right atrial fistula to prevent recurrence of the infective endocarditis.

**Fig. 3:**
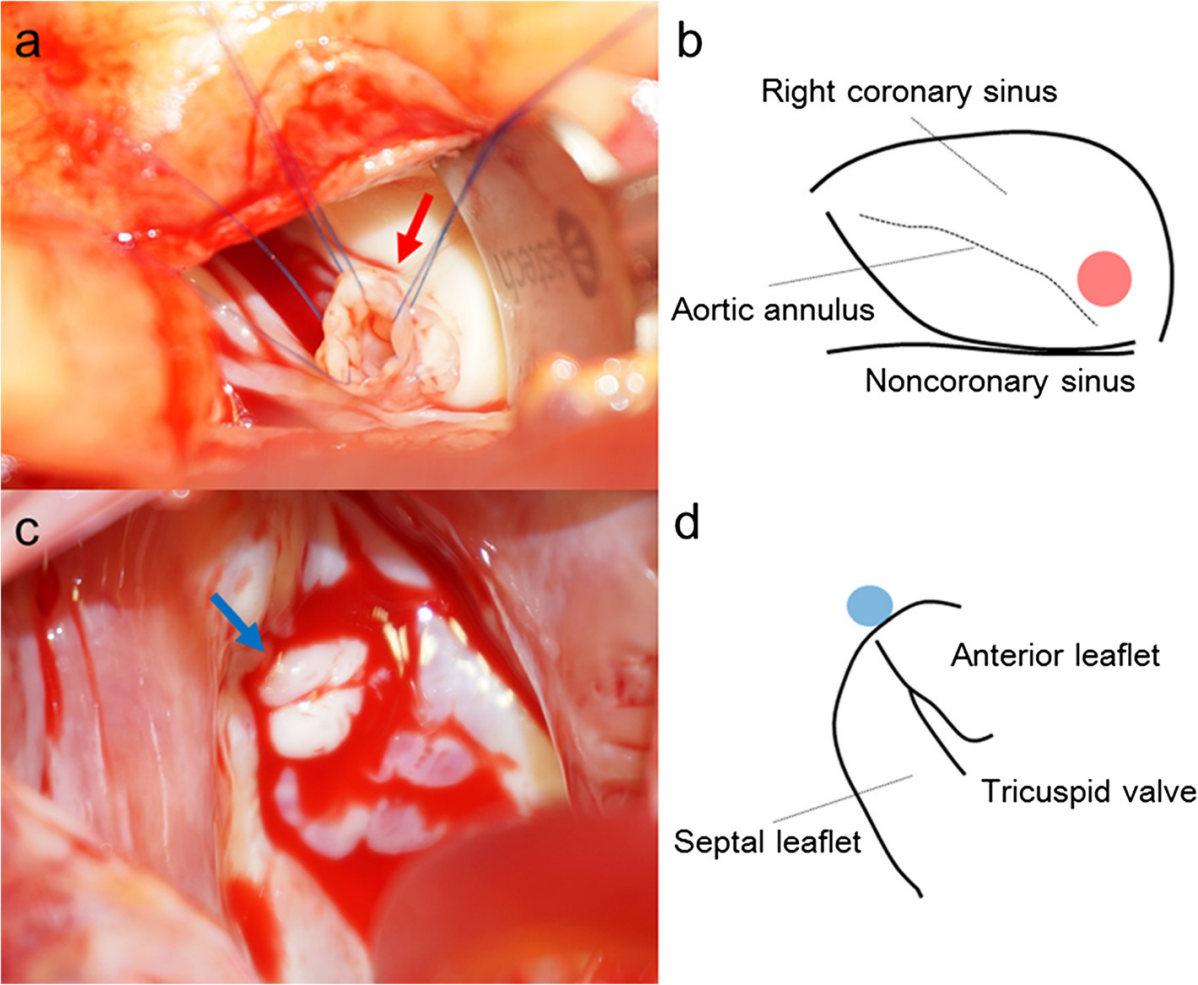
**a** An intraoperative photograph showing the windsock-like aneurysm being pulled into the sinus of Valsalva (*red arrow*). **b** A schema showing the position of the aneurysm, which is located just above the aortic annulus of the right coronary cusp (*red circle*). **c** An intraoperative photograph showing the exit into the right atrium, which was surrounded by thickened excessive tissue (*blue arrow*). **d** A schema showing the position of the exit into the right atrium, which is located near the anteroseptal commissure of the tricuspid valve, which corresponds with the membranous septum (*blue circle*)

The surgery was performed through a median sternotomy. Cardiopulmonary bypass was established by aorto-bicaval cannulation. After clamping the ascending aorta, selective cardioplegia was administered. Through an aortotomy, we observed a fistula, which was opened just above the aortic annulus of the right coronary cusp. A windsock structure, which had a diameter of 5 mm, was pulled out from the fistula into the sinus of Valsalva. We did not observe any vegetation at the tip of the fistula, but the wall of the sinus of Valsalva aneurysm was slightly thickened (Fig. 3a, b). The exit in the right atrium was located near the anteroseptal commissure of the tricuspid valve, which corresponded with the membranous septum (Fig. 3c, d). There was no vegetation on the aortic and tricuspid valves. The opening in the sinus of Valsalva was closed using an autologous pericardial patch, whereas the exit into the right atrium underwent a direct closure. Although the exit closure into the right atrium had a risk of injury to the conduction system, we evaded the risk using the surrounding excessive tissue. The postoperative course was uneventful. Postoperative echocardiography showed no residual shunt and aortic regurgitation. One year postoperatively, no recurrence of the infective endocarditis or the L-R shunt was observed.

### Discussion

Both congenital and acquired etiologies can be involved in the development of sinus of Valsalva aneurysms. The congenital etiology relies on a structural deficiency in the aortic media, which is related to the development of the distal bulbar septum [6]. Sinus of Valsalva aneurysms are sometimes associated with congenital connective tissue diseases, including Marfan’s syndrome, Ehlers-Danlos syndrome, and Loeys-Dietz syndrome [7]. Acquired sinus of Valsalva aneurysms, which are commonly observed in middle-aged and older patients, can be caused by various etiologies, including atherosclerosis, cystic medial necrosis, infective endocarditis, syphilis, and chest trauma [8, 9].

Sinus of Valsalva aneurysms typically develop in the right coronary sinus (79 %); however, they may rarely be found in the noncoronary sinus (19.5 %) and the left coronary sinus (1.5 %) [10]. Aneurysms originating in the right coronary sinus tend to rupture into the right ventricle and atrium, whereas most aneurysms originating in the noncoronary sinus tend to rupture into the right atrium [9, 10]. Consequently, rupture of a sinus of Valsalva aneurysm results in an L-R shunt via a fistula. Most patients with rupture of a sinus of Valsalva aneurysm present with acute-onset symptoms, including palpitation and chest pain [1]. In some cases, the patients fall into acute hemodynamic collapse [7]. However, rupture of a sinus of Valsalva aneurysm is rarely asymptomatic [2, 3]. The severity of symptoms depends on the size of the shunt, the presence of other cardiac lesions, and the age at presentation [11]. The reasons for our patient being asymptomatic included the presence of a small defect (5 mm), a non-critical amount of L-R shunt (Qp/Qs 1.8), and the absence of other cardiac lesions, including the ventricular septal defect or aortic regurgitation.

In the present case, the patient was diagnosed with a heart murmur for the first time when he was 37 years old, i.e., 8 years prior to the development of endocarditis. In addition, there was no coexistence of any heart valve disease or congenital cardiac anomaly, including ventricular septal defect. Therefore, we believed that the patient had been asymptomatic for 8 years following the rupture of the sinus of Valsalva aneurysm. Furthermore, the sinus of Valsalva aneurysm was not considered to be caused by infective endocarditis. Most cases of ruptured sinus of Valsalva aneurysms due to infective endocarditis result from abscess formation following valvular endocarditis, particularly involving the aortic valve [12]. No evidence of active or healed infective endocarditis was observed in any of the heart valves in our patient. Endocarditis developing from a fistula following rupture of a sinus of Valsalva aneurysm has rarely been reported [4].

Treatment of ruptured sinus of Valsalva aneurysms is divided into open surgery and percutaneous device closure. Open surgery is conventional and includes simple plication, patch repair using autologous or bovine pericardium, and aortic root replacement [9]. The short- and long-term results of surgical repair for ruptured sinus of Valsalva aneurysms are excellent [9, 10]. Meanwhile, percutaneous device closure of a ruptured sinus of Valsalva aneurysm was first reported in 1994 [13]. This less invasive approach is being increasingly used as an alternative treatment. With regard to cases with ruptured sinus of Valsalva aneurysms treated by percutaneous device closure, failure to achieve successful closure of a large defect (>10 mm) has been previously reported [14], and the long-term outcome of this treatment is uncertain [15].

## Conclusions

We reported a case of infective endocarditis developing from an aorto-right atrial fistula caused by chronic rupture of a sinus of Valsalva aneurysm. The patient was asymptomatic for 8 years following the rupture of the sinus of Valsalva aneurysm. Although asymptomatic rupture of a sinus of Valsalva aneurysm is rare, it should be recognized as a possible cause of infective endocarditis. To prevent recurrence of infection, closure of the fistula is essential. Surgical repair is a reliable procedure for treating a rupture of a sinus of Valsalva aneurysm, particularly in cases of young and middle-aged patients.

## Consent

Written informed consent was obtained from the patient for publication of this case report and any accompanying images. A copy of the written consent is available for review by the Editor-in-Chief of this journal.

## References

[1] Dong C, Wu QY, Tang Y (2002). Ruptured sinus of Valsalva aneurysm: a Beijing experience. Ann Thorac Surg..

[2] Lee SH, Kim JB, Park NH, Kim HS, Keum DY (2013). Asymptomatic ruptured sinus of Valsalva aneurysm combined with perimembranous ventricular septal defect, and bicuspid aortic valve in adult patient. Thorac Cardiovasc Surg..

[3] Moustafa S, Mookadam F, Connelly MS (2013). Reticent uneventful rupture of right coronary sinus of Valsalva aneurysm into right ventricle. Heart Lung Circ..

[4] Medina HM, Vazquez J, Pritchett A, Lakkis N, Dokainish H (2007). Comprehensive imaging including three-dimensional echocardiography of an infected, ruptured sinus of valsalva aneurysm. Echocardiography..

[5] Hirapur I, Veeranna RM, Agrawal N. Classical windsock deformity of ruptured sinus of Valsalva: an unusual appearance on transthoracic echocardiography. BMJ Case Rep. 2014; doi:10.1136/bcr-2014-204493. 10.1136/bcr-2014-204493 PMC4039973 24862606

[6] Edwards JE, Burchell HB (1957). The pathological anatomy of deficiencies between the aortic root and the heart, including aortic sinus aneurysms. Thorax..

[7] Liu F, Zhu Z, Ren J, Mu J (2014). A rare case of sudden dyspnea and unexpected death in adolescence: fistula from aortic sinus of Valsalva to right atrium. Int J Clin Exp Med..

[8] Jugpal TS, Dixit R, Lohchab S, Garg A. Giant unruptured sinus of Valsalva aneurysm: an unusual cause of right heart failure. J Clin Imaging Sci. 2015; doi:10.4103/2156-7514.170733. 10.4103/2156-7514.170733 PMC4683788 26713180

[9] Takach TJ, Reul GJ, Duncan JM, Cooley DA, Livesay JJ, Ott DA (1999). Sinus of Valsalva aneurysm or fistula: management and outcome. Ann Thorac Surg..

[10] Wang ZJ, Zou CW, Li DC, Li HX, Wang AB, Yuan GD (2007). Surgical repair of sinus of Valsalva aneurysm in Asian patients. Ann Thorac Surg..

[11] Guo DW, Cheng TO, Lin ML, Gu ZQ (1987). Aneurysm of the sinus of Valsalva: a roentgenologic study of 105 Chinese patients. Am Heart J..

[12] Choudhary SK, Bhan A, Sharma R, Airan B, Kumar AS, Venugopal P (1997). Sinus of Valsalva aneurysms: 20 years’ experience. J Card Surg..

[13] Cullen S, Somerville J, Redington A (1994). Transcatheter closure of a ruptured aneurysm of the sinus of Valsalva. Br Heart J..

[14] Zhang B, Sun Y, Wu J, Zhu JY, Cao R, Liu XL (2015). Failed transcatheter closure of a giant ruptured sinus of Valsalva aneurysm. Chin Med J..

[15] Kuriakose EM, Bhatla P, McElhinney DB (2015). Comparison of reported outcomes with percutaneous versus surgical closure of ruptured sinus of Valsalva aneurysm. Am J Cardiol..

